# Editorial: Coping with pollution - the effects of environmental contaminants on plant growth and physiology, volume II

**DOI:** 10.3389/fpls.2023.1131656

**Published:** 2023-01-25

**Authors:** Marcelo Pedrosa Gomes, Eduardo Gusmão Pereira, Bao-Sheng Qiu, Philippe Juneau

**Affiliations:** ^1^ Laboratório de Fisiologia de Plantas sob Estresse, Departamento de Botânica, Setor de Ciências Biológicas, Universidade Federal do Paraná, Curitiba, Paraná, Brazil; ^2^ Laboratório de Fisiologia do Estresse Abiótico, Instituto de Ciências Biológicas, Universidade Federal de Viçosa, Florestal, Minas Gerais, Brazil; ^3^ School of Life Sciences, and Hubei Key Laboratory of Genetic Regulation and Integrative Biology, Central China Normal University, Wuhan, Hubei, China; ^4^ Ecotoxicology of Aquatic Microorganisms Laboratory, EcotoQ, GRIL, TOXEN, Department of Biological Sciences, Université du Québec à Montréal, Montréal, Montréal, QC, Canada

**Keywords:** trace elements, chemicals, climate changes, phytoremediation, efficiency

Although environmental contamination by metals is an old concern and despite the number of studies addressing that problem, investigations aiming at understanding toxicological responses as well as the improvement of nature-based solutions to recover metal-impacted ecosystems are still needed. The planet has been faced with intensified anthropogenic activities, such as excessive disposal of mining waste and industrial and agricultural effluents. These contamination sources are responsible for the insertion of tons of toxic metals to ecosystems; events demanding appropriate management actions. One of the most feasible solutions to reclaim soils polluted with toxic metals is the use of plants in phytoremediation technique. Although phytoremediation has proven as a green technology for metal removal from low-to-moderate contaminated sites, the selection of plant species as well as of supporting strategies aiming to enhance phytoremediation efficiency merit attention (Gomes et al., 2021).

Due to their high biomass yields, fast growth, adaptation strategies to infertile soils and successive short regrowth after harvest, grasses have been proposed as prospective candidates for metal phytoremediation. In their review, Rabêlo et al. synthesized information on mechanisms involved in uptake, accumulation and tolerance to toxic metals, identified suitable grass species for phytoremediation processes, described strategies used to improve their phytoremediation efficiency and, mainly, pointed out to the advantages and disadvantages of using grass species in phytoremediation. Although grasses generally have great tolerance to metals, there are sensitive to adverse climate conditions (such as water deficit and low temperatures), there are mostly effective to withdrawing metals from the topsoil due to poor root penetration in the substrate and, finally, several species are particularly sensitive to metal pollutant mixtures, therefore, compromising their phytoremediation efficiency. As previously stated by Gomes et al. (2021), the interactive effects of different contaminants with unprecedent variable environmental factors linked to the Climate Changes may constrain phytoremediation programs.

In that context, Kang et al. investigated the use of biochar passivation as an economical technology to prevent contamination or to be used as a support for phytoremediation purposes. The authors observed that biochar application significantly alleviated the phytotoxicity of Cd and Zn to foxtail millet and increased the abundance and richness of soil microorganisms, showing that biochar is an effective agent for the remediation of metal-contaminated soils. Similarly, Zheng et al. investigated the potential of salicylic acid (SA) to improve the Cd-phytoremediation capacity of aquatic metal-accumulator plants. By modulating physiological responses, including the antioxidant system, SA promoted the growth and improved the phytoextraction capacity of *Nasturtium officinale* and may constitute a strategy to be employed on phytoremediation programs.

In addition to the use of alternative tools to improve phytoremediation, Tartaglia et al. highlighted the importance to understand plants, fungi and soil bacteria as a meta-organism that reacts together to environmental stimuli. The authors evaluated the potential of a microbial consortium along with a known phytoremediatior species (*Schedonorus arundinaceus*) to extract metals, polycyclic aromatic hydrocarbons (PAHs) and polychlorinated biphenyls (PCB) from pluricontaminated soils. The results indicate the complex interactions occurring at the rhizosphere after the biotechnological treatment which was related to phytoremediation capacity of plants.

We are grateful to all contributors for presenting such a variety of aspects of different complexity levels from molecular to physiological responses of plants to contaminants. It also became clear the emerging role of alternative agents such as biochar passivation and the use of chemicals known to increase plant performance to improve metal-phytoremediation efficiency. Moreover, it was highlighted the importance of considering tripartite enzymatic activity between plants, bacteria and fungi in the rhizosphere to understand and improve phytoremediation programs. The contributions from this Research Topic also highlighted the remaining knowledge gaps, among them are (not exclusive):

1. The interactive effects of different contaminants on plants.
2. The interactive effects of contaminants and changes in the environment arising from Climate Change (such as increasing temperatures, irregular rain patterns, and adverse light conditions).
3. The use of chemicals as alternative for enhancing phytoremediation capacity of plants.

In conclusion, priorities for future work on phytoremediation should focus on investigating isolated and combined effects of contaminants with other chemicals and environmental abiotic and biotic conditions, which may play important roles in enhancing phytoremediation capacity of plants.

## Author contributions

All authors listed have made a substantial, direct and intellectual contribution to the work, and approved it for publication.
